# Analysis of structural variants in four African cichlids highlights an association with developmental and immune related genes

**DOI:** 10.1186/s12862-020-01629-0

**Published:** 2020-06-22

**Authors:** Luca Penso-Dolfin, Angela Man, Tarang Mehta, Wilfried Haerty, Federica Di Palma

**Affiliations:** Earlham Institute, Norwich Research Park, Colney Lane, Norwich, NR47UZ UK

**Keywords:** Adaptation, Genome evolution, Structural variants

## Abstract

**Background:**

East African lake cichlids are one of the most impressive examples of an adaptive radiation. Independently in Lake Victoria, Tanganyika, and Malawi, several hundreds of species arose within the last 10 million to 100,000 years. Whereas most analyses in cichlids focused on nucleotide substitutions across species to investigate the genetic bases of this explosive radiation, to date, no study has investigated the contribution of structural variants (SVs) in the evolution of adaptive traits across the three Great Lakes of East Africa.

**Results:**

Here, we annotate and characterize the repertoires and evolutionary potential of different SV classes (deletion, duplication, inversion, insertions and translocations) in four cichlid species: *Haplochromis burtoni, Metriaclima zebra, Neolamprologus brichardi* and *Pundamilia nyererei*. We investigate the patterns of gain and loss evolution for each SV type, enabling the identification of lineage specific events. Both deletions and inversions show a significant overlap with SINE elements, while inversions additionally show a limited, but significant association with DNA transposons. Inverted regions are enriched for genes regulating behaviour, or involved in skeletal and visual system development. We also find that duplicated regions show enrichment for genes associated with “antigen processing and presentation” and other immune related categories. Our pipeline and results were further tested by PCR validation of selected deletions and inversions, which confirmed respectively 7 out of 10 and 6 out of 9 events.

**Conclusions:**

Altogether, we provide the first comprehensive overview of rearrangement evolution in East African cichlids, and some important insights into their likely contribution to adaptation.

## Background

African cichlids represent one of the best examples of rapid adaptive radiation [1–4]. The adaptation to different ecological niches in Lakes Malawi, Tanganyika and Victoria has given rise to several hundreds of species in a period of just a few million years [5–7]. The radiation is associated with great phenotypic variation, including jaw morphology, body shape, coloration, adaptation of the visual system to different water depths, and behavior [8–16]. Variation in ecological niches and behaviour appears to be associated with different brain development [17], with differences appearing already at early developmental stages [18]. A great example of adaptation is represented by the evolution of the cichlid visual system, involving eight different opsin genes [19–21].

To gain insights on the molecular mechanisms underlying this rapid radiation, Brawand et al. [22] generated genome references for five species: the Nile tilapia *(Oreochromis niloticus),* representing an ancestral lineage; *Neolamprologus brichardi* (Lake Tanganyika), *Metriaclima zebra* (Lake Malawi), *Pundamilia nyererei* (Lake Victoria), and *Haplochromis burtoni* (riverine species around Lake Tanganyika). The study highlighted several mechanisms underlying species diversification, including selection acting on existing standing variation, high rates of gene duplication, novel microRNAs and rapid sequence divergence in otherwise conserved non-coding elements. Following this study, Malinksy et al. described an example of early stage divergence between two cichlid ecomorphs in Tanzania [8]. They identified genomic islands of speciation between them, containing potentially adaptive genes associated with mate choice. Theis et al. [23] focused on the early phases of adaptive divergence of *H. burtoni,* which is found in both Lake Tanganyika and inflowing river. Their results highlighted the presence of multiple divergent lake-stream populations, representing different stages of the speciation process. More recently, the sequencing of 134 individuals covering 73 species provided a great characterisation of genomic diversity in lake Malawi [24]. The authors observed very low levels of inter-species divergence (0.1–0.25%), overlapping the diversity found within species. Phylogenetic analyses showed that no single species tree can efficiently represent all species relationships, suggesting high levels of repeatedly occurring gene flow.

In 2014, Fan and Meyer [25] used the five genome references generated by Brawand et al. [22] to annotate SNPs, indels and SVs in four of these species, representative of the adaptive radiations. However, this study applies one method (Pindel v0.2.5a1) of detecting SVs based on a less complete and contiguous Nile tilapia assembly (*Orenil1.1*) than the available PacBio reference genome [26, 27], and does not focus on the adaptive potential of large-scale variation.

Recently, Conte et al. generated an improved reference assembly for the cichlids *M. zebra* and *O. niloticus* [26]. The authors compared the genome structure of the two species at the chromosome scale, taking advantage of the high quality of these references. They observed a high number of ~ 2-28 Mb, intra-chromosomal SVs, but a limited number of inter-chromosomal rearrangements. They also identified structural changes associated with lower recombination rates, suggesting inversion events between different species in Lake Malawi. This study, however, did not investigate the patterns of SV evolution across representative cichlid genomes of the three East African Great Lakes, or consider their possible implication in speciation and adaptive phenotypes.

All other studies so far focused on single variation within and between species and to a lesser extent on the evolution of gene regulatory patterns [27]. Structural variants (SVs, including deletions, duplications, inversions, insertions and translocations) are the source of increased genomic variability and in some cases adaptive potential. Gene or exon duplication events might lead to neo- or sub-functionalisation [28–32]. An evolutionary study in East African cichlids focusing on *agrp2* (a locus controlling horizontal stripe patterns) revealed several recent duplications, insertions, and deletions, including a tandem duplication of the last exon [33]. This event is not fixed in any of the radiations, and is polymorphic even within some species. This pattern of copy number variation can facilitate neofunctionalization or even loss-of-function of *agrp2*.

Gene loss events, on the other hand, can reflect relaxed selective pressure or be possibly adaptive in other cases [34]. For example, the loss of *ampd3* in sperm whales likely represents an adaptation to their extreme diving ability [34, 35].

Inversions result in suppressed recombination when heterozygous, and might act as a protection against gene flow for specific haplotypes [36]. Inversions might raise in frequency, up to fixation, possibly leading to isolation and even speciation events [37, 38]. Studies in *Drosophila melanogaster* provided strong evidence for their involvement in adaptation. For example, the inversion 3RP is associated with adaptation to different climates [39]. Its frequency exhibits a parallel latitudinal cline across several continents, being higher close to the equator and decreasing towards higher latitudes [38, 39]. Translocations can result in a heavy restructuration of chromosome organisation [40], with potential gene loss or changes in regulatory control of expression.

Identifying structural changes across species representative of all three great lakes can provide exciting insights into their explosive radiation. In this study, we use the newly released *O. niloticus* (riverine species living in shallow waters) reference based on long read PacBio sequencing [41] and paired-end sequencing data generated by [22] to identify SVs in four representative cichlid species with varying ecological adaptations: *Neolamprologus brichardi* (Lake Tanganyika, reef dwelling planktivore, 3–30 m of water depth), *Metriaclima zebra* (Lake Malawi, rock dwelling algae scraper, 6–28 m of water depth), *Pundamilia nyererei* (Lake Victoria, reef dwelling planktivore, 4–7 m of water depth), and *Haplochromis burtoni* (insectivorous riverine species around Lake Tanganyika). Through this analysis we aim to: characterise the evolutionary patterns associated with different rearrangement classes; investigate functional enrichment within those rearranged genomic regions; identify the genes affected by these structural changes and how these can relate to the phenotypes found across the three lakes.

We show that genes lying inside inverted regions are enriched for genes regulating behaviour, or involved in skeletal and visual system development, which are directly relevant to the African radiation. Altogether, we describe the repertoires of structural variations across four species of the East African cichlids, their evolutionary dynamics, and novel insights into their possible contribution to adaptation.

## Results

### Annotation of SVs across 4 cichlid species

We mapped all previously generated paired-end libraries [22] to the high quality *O. niloticus* assembly [41] to annotate five different classes of rearrangements (deletion, tandem duplication, inversion, insertion and translocation) in the four available species of the East African radiation (Supplementary Fig. 1). We used a combination of three different tools: Breakdancer [42], Delly [43] and Pindel [44] and identified 6694 deletions, 1550 duplications, 1471 inversions, 34,875 insertions and 1354 translocations (Table 1, Additioal file 2: Supplementary File 1).

**Table 1:** List of annotated SVs

SV class	Total	> 1 species	All species
DEL	6694	3903	541
DUP	1550	353	22
INV	1471	566	188
INS	34,875	18,253	3949
TRA	1354	92	2

**Fig. 1:**
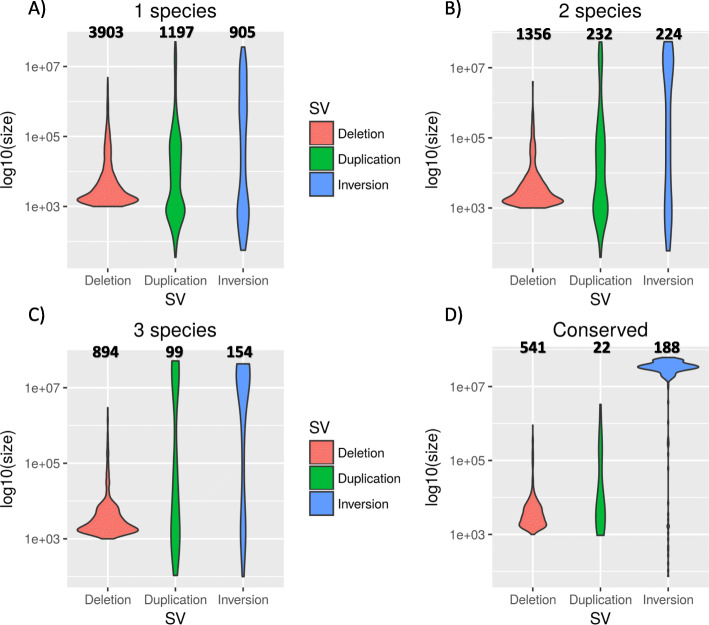
Violin plots of the size of different intra-chromosomal SV classes, considering different levels of conservation (“conserved” refers to SVs common to all 4 species)

Our initial predictions showed a bias towards small (< 1 kb) deletions (240229). This number might be inflated as a result of our SV detection pipeline, where deletions are identified using read pairs mapped in a concordant way (as opposed to duplications and inversions). This represents an issue particularly when considering small events. Therefore, we decided to retain only deletions with a minimum size of 1 kb (Table 1). In the resulting dataset, 5483 deletions fall in the 1–10 kb size range, while 1207 represent larger, > 10 kb events (Fig. 1). We investigated whether the size of a SV correlates with the age of the event. While the size distributions of deletions did not seem to be affected by the number of species sharing the SV, we noticed a tendency for duplications and inversions towards larger sizes as the number of species increased. Species specific events are significantly smaller than those common to 2 species (MW test, *p* = 0.005), which in turn are smaller than the events found in 3 species (MW test; duplications: *p* = 0.008; inversions: *p* < 0.0001; see Additional file 3: Supplementary File 2). Moreover, conserved inversions are significantly larger than both conserved deletions (MW test, *p* < 0.0001) and duplications (MW test, *p* < 0.0001).

**Fig. 2:**
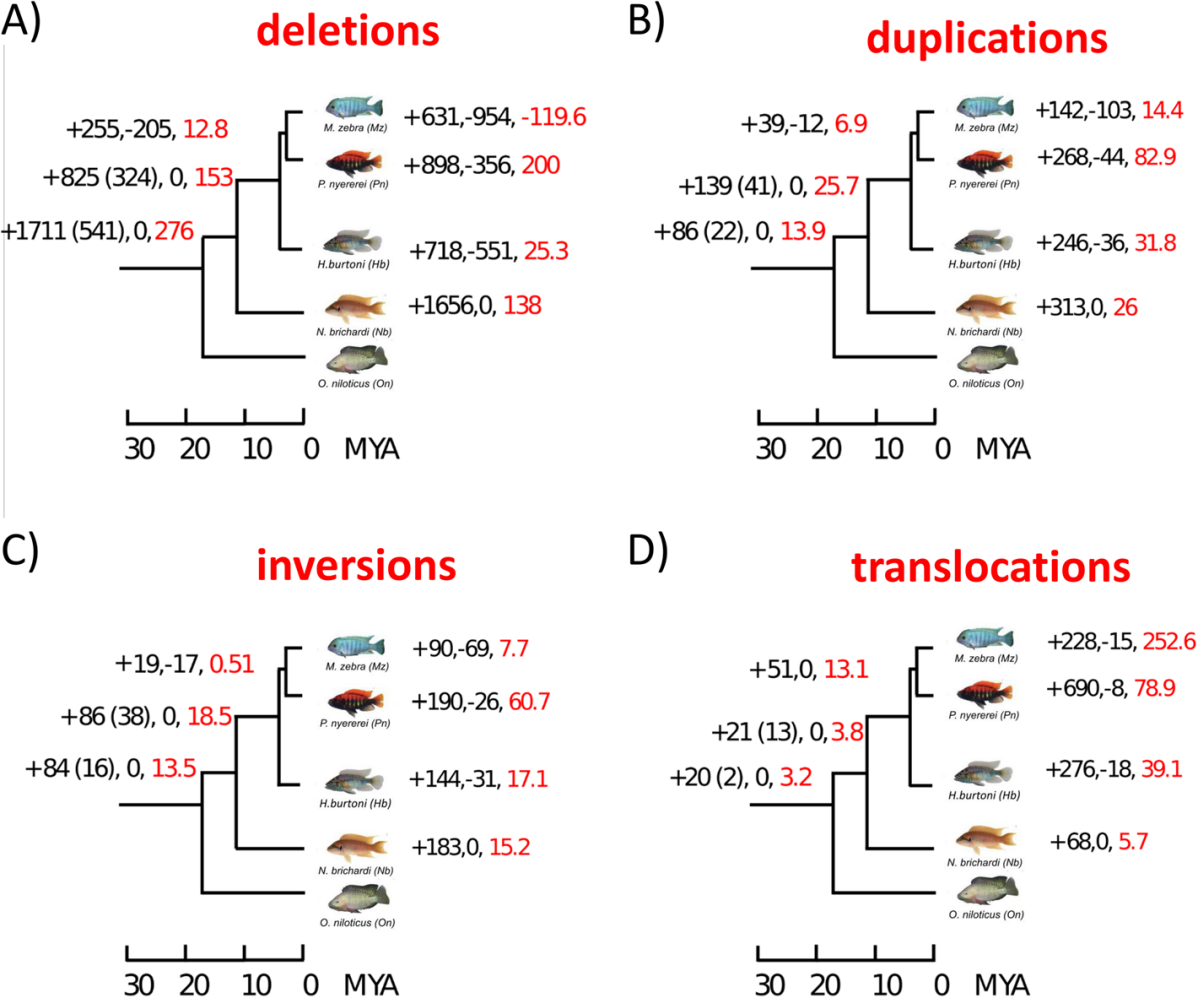
Gain and loss of different SV types (up to 5 Mb in size) across the phylogenetic tree. For each branch, the number of gained and lost events is provided, as well as the net gain rate per million years (red labelled). When different from the total number of gains, the number of events which are gained and retained across the whole lineage (not lost afterwards) is indicated in brackets

We investigated the patterns of gain and loss evolution for each SV class, using a Dollo Parsimony approach (see Methods). We identified a high proportion of events predicted to be lineage specific (Fig. 2). Additionally, comparison across species allowed us to identify the events common to a single lineage or to all species involved in the African radiation. We will refer to the latter as “conserved SVs”. However, a “conserved SV” could also represent a structural change that occurred in the *O. niloticus* lineage, and this ambiguity cannot be resolved without the addition of an outgroup species.

We noticed a surprisingly high loss rate of deletions in the *M. zebra* lineage (Fig. 2a). In order to evaluate the reliability of our approach and the accuracy of our annotations, we compared our results to those obtained through the pairwise, whole genome alignments between the latest *M. zebra* and *O. niloticus* assemblies, using *Satsuma2* (https://github.com/bioinfologics/satsuma2, see Methods). Out of 2263 deletions annotated in *M. zebra*, only 54 (2%) were discordant with Satsuma2 alignments. Thus, we show that our annotation in *M. zebra* has a very high concordance with the high quality genome assemblies of *M. zebra* and *O. niloticus*.

With the exception of *M. zebra* deletions, we observed high proportions of lineage specific events, consistent across all SV classes (Fig. 2). However, in the case of deletions we also observed a high number (1711) of events which are ancestral to the radiation. Overall, these results point at a reduction in genome size associated to the African radiation. While this is in concordance with the observation that the *M. zebra* assembly is 48 Mb shorter than the *O. niloticus* reference [26, 41], conserved SVs might also reflect a rearrangement event specific to *O. niloticus*, as stated previously.

We investigated the extent of interval overlap between our predicted SVs and different genomic features. We considered different subsets of our SV annotations, categorising our predictions based on size range (< 1 kb, 1 kb–10 kb,> 10 kb) and number of species sharing the SV event (whole dataset vs conserved SVs). We observed a strong association between > 10 kb conserved deletions and immunoglobulin chain regions. The association is highly significant for both the constant (14.8 fold change, *p* = 0.01) and variable (10.8 fold change, *p* = 5e-03) gene segment annotation, which suggests a possible involvement of copy number variants in immune response mechanisms. It must be pointed out, nevertheless, that these loci are present in multiple, tandemly repeated copies, and the observed association could possibly reflect assembly issues in repetitive regions.

We also hypothesised that repeats throughout the genome facilitate the evolution of structural changes. In order to test this hypothesis, we looked at the genomic association (interval overlap analysis, see Methods) between our SV dataset and African cichlid specific repetitive elements. These analyses highlighted a significant overlap between 1 kb–10 kb long conserved inversions and SINE2 elements (10.2 fold change, *p* = 8.7e-03). The association with SINE2 is not significant, however, when we consider all conserved inversions, irrespective of their size (1 fold change, *p* = 0.3).

Conserved duplications are significantly under-represented with African cichlids (AFC) SINE2–1 (0.64 fold change, *p* = 2.7e-03) and REX1–2 AFC elements (0.3 fold change, *p* = 3.14e-02). Conversely, they appear to be enriched for several simple repeats, including (AAGTCTC) n (54.7 fold change, *p* = 1e-04).

Large deletions appear to be negatively associated with AFC RTE-2 elements (0.57 fold change, *p* = 3.7e-02) but positively associated with AFC L1–1 elements (2.17 fold change, *p* = 8.7e-03), as well as several simple repeats. When we considered all conserved deletions, irrespective of their size, we observed a significant association with AFC SINE2–1 (1.42 fold change, p = 1e-04) and SINE3 (2.98 fold change, p = 1e-04) elements. Similar conclusions were reached in previous studies on the pig genome [45, 46]. Taken together, these results suggest a correlation between repetitive elements and structural evolution in African cichlids.

We next asked the question whether we observe differences in the repeat landscape inside and outside SV regions. In order to identify homologous regions between the reference and each of the remaining species, we converted *Satsuma2* whole genome alignments to chain format, and performed a liftover of all SV coordinates from the reference to each of the other species. This allowed us to compare the repeat landscape in a pairwise fashion, considering different SV class separately.

**Fig. 3:**
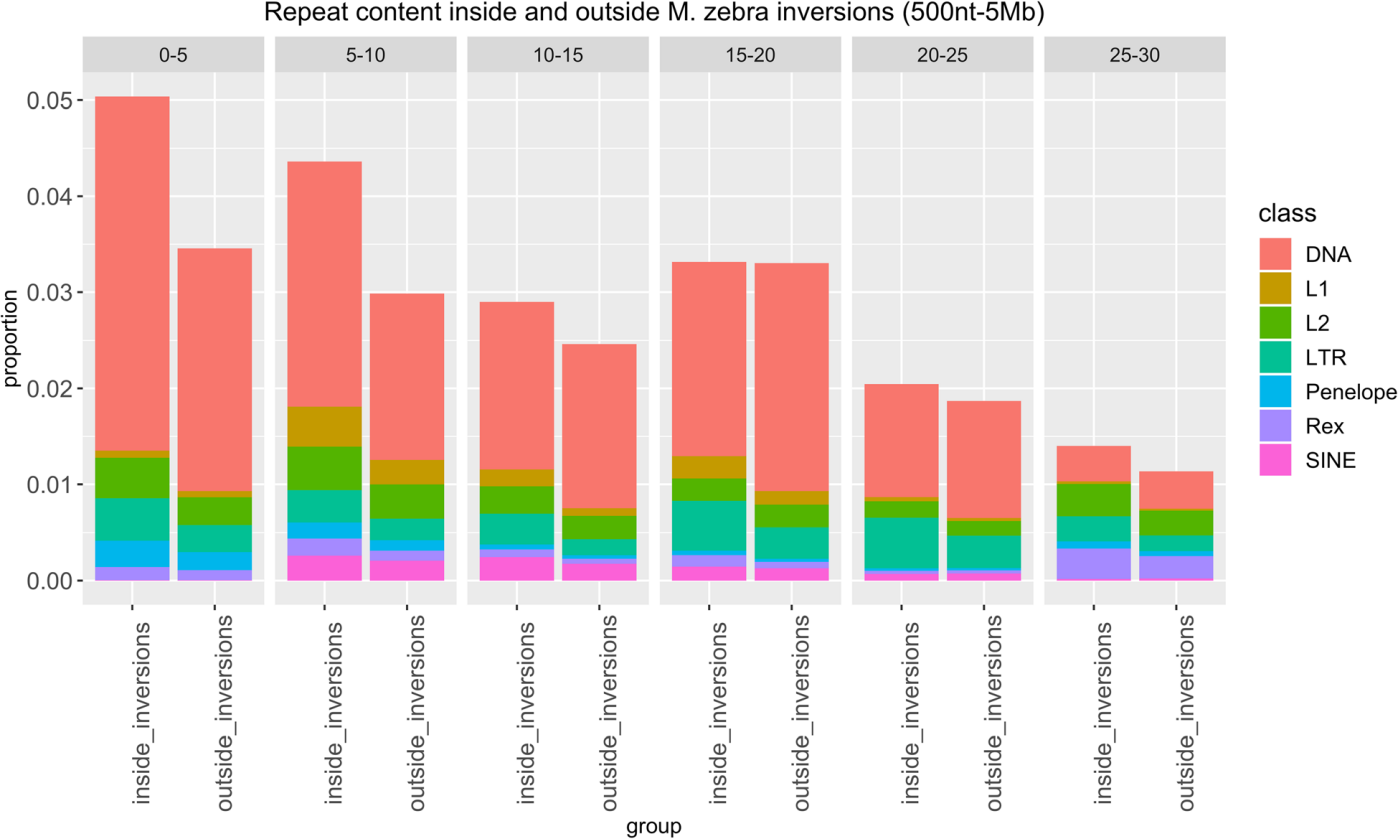
Proportion of nucleotides inside and outside *M. zebra* inversions which are part of a repeat element, grouped based on the percentage of divergence from the consensus. Different colours correspond to distinct repeat classes. Each grid corresponds to a specific divergence interval

**Fig. 4:**
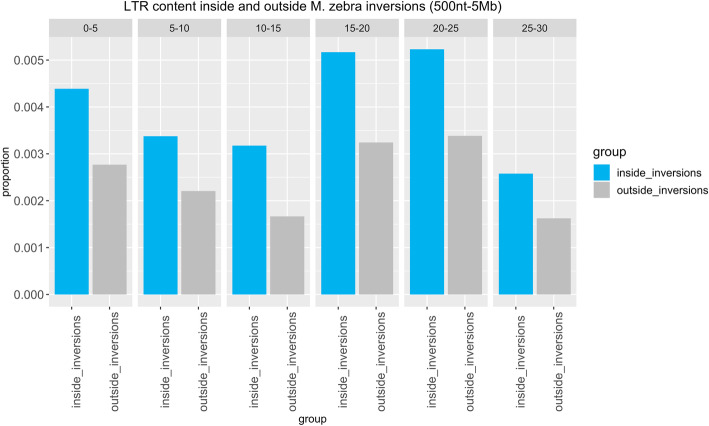
Proportion of nucleotides inside and outside *M. zebra* inversions which are part of an LTR elements, grouped based on the percentage of divergence from the consensus

**Fig. 5:**
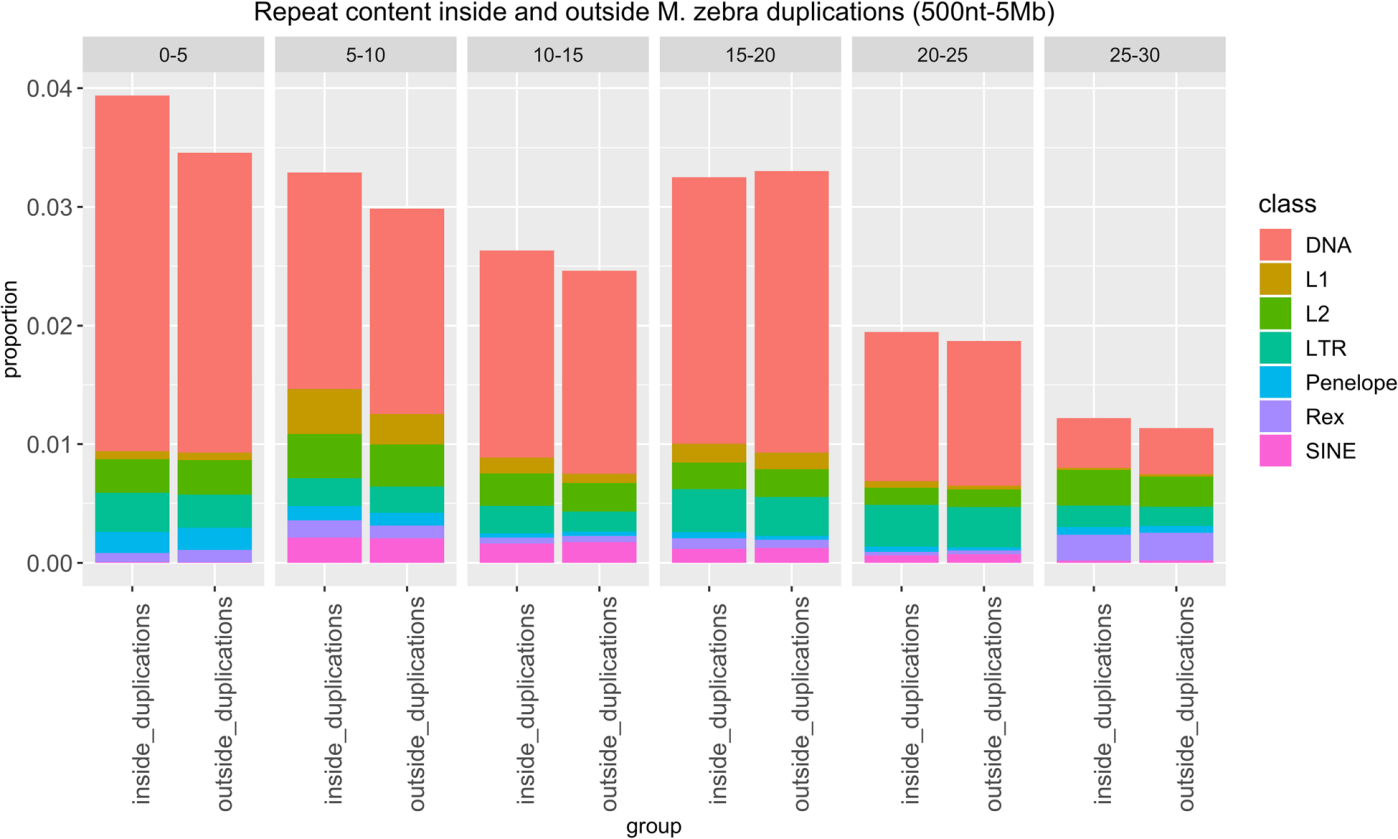
Proportion of nucleotides inside and outside *M. zebra* duplications which are part of a repeat element, grouped based on the percentage of divergence from the consensus. Colours and categories are defined as in Fig. 3

When heterozygous, an inversion can favor the accumulation of mutations and novel transposable elements, as a result of reduced excisions rates [47, 48]. We tested this possibility by comparing the repeat content inside and outside inverted regions. We focused our analysis on the latest *O. niloticus* genome reference. Previous studies highlighted very high proportions of DNA transposable elements in African cichlids [22], an observation which was confirmed by our data (Figs. 3, 4 and 5; SVs annotated in *M. zebra*). Overlap analyses based on the *O. niloticus* reference suggested a limited, but significant enrichment in DNA transposons inside inversions (size range: 500 nt-5 Mb; fold change=1.07, *p*= 1e-04). While for other repeat classes the proportions are very similar inside and outside inverted regions (Fig. 3), the LTR representation is higher in the former across all divergence bins (Fig. 4). This reflected in a significant enrichment in LTR elements inside inversions (size range: 500 nt-5 Mb; fold change= 1.21, *p*= 1e-04), as opposed to the LTR content outside inversions (fold change=0.92, p= 1e-04).

Next, we repeated the analyses considering duplication events. We observed no difference in repeat content inside and outside duplicated regions (Fig. 5). The association (based on the *O. niloticus* reference sequence) between duplications and LTR is weaker than expected (fold change = 0.94, *p* = 1e-04), while no significant deviation was found when considering DNA transposons (fold change = 0.99, *p* = 0.31). As for regions outside duplications, we observed a significant, although very limited, enrichment for both DNA (fold change = 1.001, *p* = 0.04) and LTR (fold change = 1.05, *p* = 8e-04) elements. It must be noted, however, that during the liftover conversion of the genomic coordinates, many inverted and duplicated regions were lost, limiting the sequence space considered in *M. zebra*.

### SV regions are enriched for developmental and immune related genes

Structural variation can provide important evolutionary novelty for speciation and the evolution of adaptive traits [28–30, 37]. For instance, gene duplication can lead to dosage effects, neofunctionalisation or subfunctionalisation events (Lynch 2002; Katju and Lynch 2006), while inverted regions can experience drastically reduced recombination rates [37]. We took advantage of our SV dataset across 4 species, to investigate which genes are affected by duplication or inversion events. We first considered different subsets of inversions, separating species specific events from the ones annotated in multiple species. When looking at species specific events, we considered each species separately (Additional file 4: Supplementary File 3). We identified 559 genes in *H. burtoni*, 109 in *M. zebra*, 580 in *N. brichardi* and 814 in *P. nyererei*. Results for *H. burtoni* highlighted GO:0006955 (“immune response”, significant: 13; expected: 5.01; p_adj_= 0.0015) and GO:0007600 (“sensory perception”, significant: 10; expected: 5.04, p_adj_= 0.03).

Inverted genes in *N. brichardi*, are enriched for GO:0065007 (“biological regulation”, significant: 193; expected: 154.35, p_adj_ = 0.0411) and GO:0009416 (“response to light stimulus”, significant: 6; expected: 2.45, p_adj_ = 0.0351). Interestingly, we also found one gene (*gja3*, coding for an intercellular channel) annotated to GO:0048050, (“post-embryonic eye morphogenesis”). *P. nyererei* genes are enriched for GO:0007602 (“phototransduction”, significant: 6, expected: 1.44, p_adj_ = 0.003). This set also includes 2 genes annotated to GO:0002089 (“lens morphogenesis in camera-type eye”, significant: 2, expected: 0.23, p_adj_ = 0.018), *fn1a* and *foxe3*, as well as 3 genes annotated to GO:0061035 (“regulation of cartilage development”, significant: 3, expected: 0.54, padj =0.015): *sox32*, *s1pr2* and *pthlha*. Members of the *sox* gene family encode for transcription factors, and play a crucial role in morphological and behavioural variation in teleosts [49]. *pthlha* is an oral jaw specific gene [50] coding for the parathyroid hormone. In the case, of *M. zebra*, we could only identify one accession represented by 5 or more genes: GO:0006468 (“protein phosphorylation”; significant: 6; expected: 2.52;; p_adj_ = 0.0374).

**Fig. 6:**
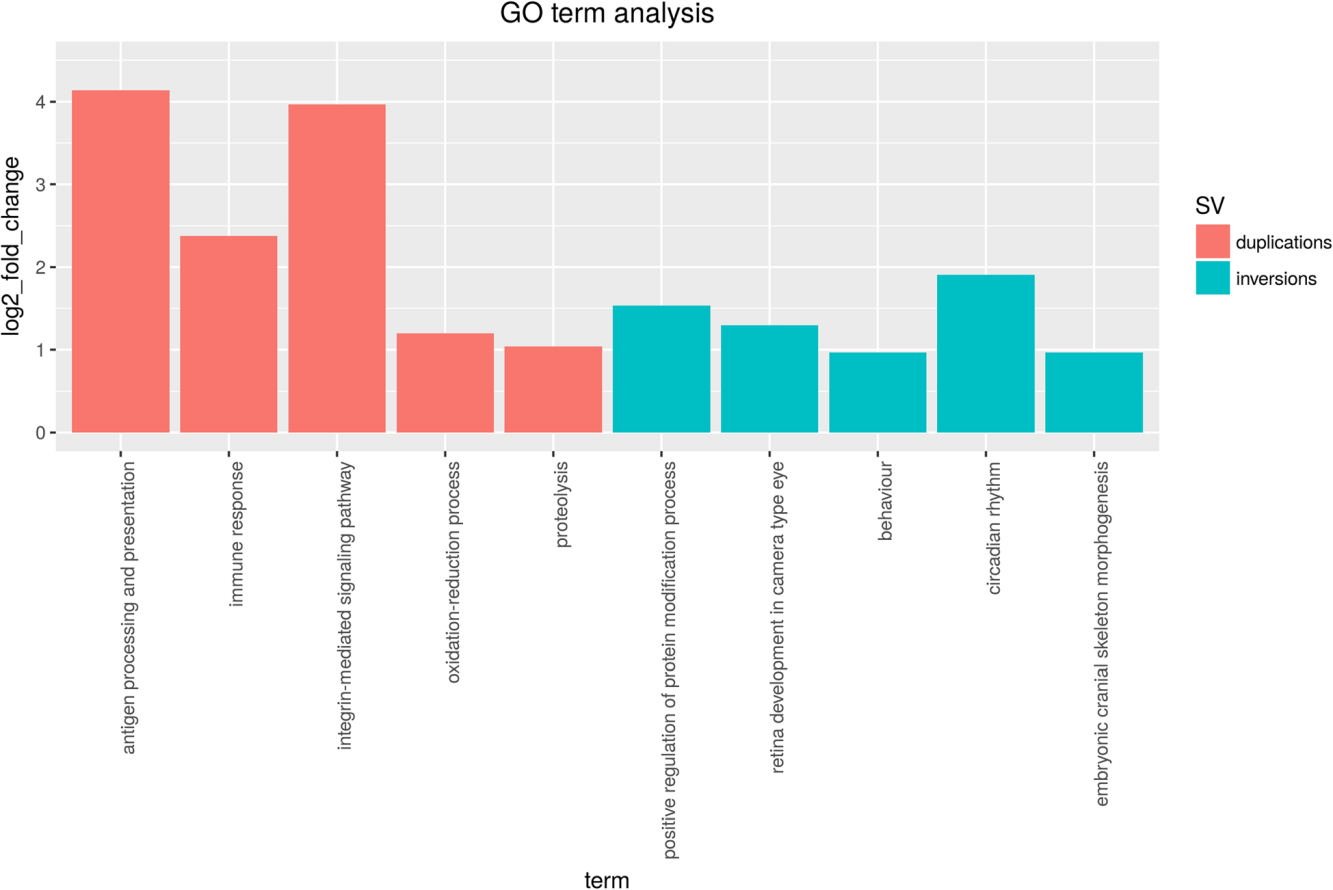
Selected GO terms found to be significantly enriched for the gene sets inside multi-species duplication and inversion (up to 5 Mb) events

We also considered inversions which are common to at least 2 species,not exceeding 5 Mb in size (Fig. 6). A total of 854 GO annotated genes (Additional file 4: Supplementary File 3) could be identified inside these SV regions. GO:term enrichment on this gene set highlighted accessions GO:0007610 ("behavior", significant: 9; expected: 4.66; p_adj_=0.042), GO:0060041 (“retina development in camera-type eye”, significant: 14; expected: 5.7; p_adj_ = 0.001), GO:0060042 (“retina morphogenesis in camera-type eye”, significant: 7; expected: 2.76, p_adj_ = 0.0018), GO:0048706 (“embryonic skeletal system development”, significant 10; expected: 5.12; p_adj_ = 0.03). Among the genes annotated to GO:0060041, we found: *vax2* (Ventral Anterior Homeobox 2), a gene known to regulate cone opsin expression [51]; *fgf8a* (fibroblast growth factor 8a), part of a key pathway in animal evolution [52], and *ift172* (intraflagellar transport 172).

We repeated the same procedure for inversions up to 10 Mb in length, which increased the number of genes considered to 1404 (Additional file 4: Supplementary File 3). While accession GO:0060041 was still significantly over-represented, we observed additional, immune related processes: GO:0019882 (“antigen processing and presentation”, significant: 8; expected: 2.7; p_adj_ = 0.0044), GO:0006955 (“immune response”, significant: 19; expected: 12; p_adj_ = 0.03) and GO:0042445 (“hormone metabolic process”, significant: 6; expected: 2.14; p_adj_ = 0.01). As part of GO:0006955, we found the gene *nfil3* (Nuclear Factor, Interleukin 3 Regulated), coding for a transcriptional regulator.

Next, we compared the GO terms across different subtrees of our five-species phylogeny (Supplementary Fig. 2, Additioal file 4: Supplementary File 3). We first selected inversions common to *M. zebra* and *P. nyererei* but absent in the other species, for which we could identify 210 inverted genes. We found enrichment for protein modification and processing, including GO:0006508 (“proteolysis”, significant: 14; expected: 7.3; p_adj_ = 0.014). When considering the branch leading to these two species as well as *H. burtoni* (134 genes, events absent in *N. brichardi*), we identified genes involved in developmental processes, including 4 annotated to GO:0048598 (“embryonic morphogenesis”) and 2 genes for accession GO:0033339 (“pectoral fin development”): *cyp26c1* (cytochrome P450, family 26, subfamily C, polypeptide 1) and *sall4* (Spalt Like Transcription Factor 4)*.* The former lies in a sex associated region in *H. burtoni* [53]. Together with the fact that the inversion is lineage specific, it makes the gene particularly interesting. We can speculate that the inversion event might have helped the maintenance of specific haplotypes (including gene *cyp26c1*) through the suppression of recombination in the affected region, possibly contributing to the divergence of sex-associated traits.

The enrichment for developmental processes was also observed for genes in conserved inversions (up to 5 Mb in size, *n* = 90), among which GO:0060042 (“retina morphogenesis in camera-type eye”, < 5 genes) and GO:0048706 (“neuron development”, significant 5; expected: 1.3; p_adj_ = 0.04) are particularly interesting.

We also looked for genes contained inside tandem duplications, and filtered the resulting set based on evidence of tandem repeat of at least 3 consecutive exons in the target genome assembly (see Methods). When considering species-specific events (Additioal file 4: Supplementary File 3), we identified 204 genes in *H. burtoni*, 197 in *M. zebra*, 143 in *N. brichardi* and 224 in *P. nyererei*. For the *H. burtoni* gene set we identified, among others, GO:0006508 (“proteolysis”, significant: 19; expected: 7.3; p_adj_ = 0.021) and GO:0060078 (“regulation of postsynaptic membrane potential”, significant: 6; expected: 0.87; p_adj_ = 0.0002). Duplicated genes in *P. nyererei* are enriched for immune related processes, including GO:0006955 (“immune response”, significant: 10, expected: 1.83; p_adj_ = 1.4e^− 5^) and GO:0019882 (“antigen processing and presentation”, significant: 6; expected: 0.41, p_adj_ < 0.0001). Additionally, GO:0055085 is represented by 18 genes (“transmembrane transport”, significant: 18; expected: 11.18, p_adj_ = 0.028).

By requiring the duplication event to be shared by at least 2 species (Fig. 6), we could identify 152 genes (Additional file 4: Supplementary File 3). Results highlighted the presence of GO:0019882 (“antigen processing and presentation”, p_adj_ = 1e^− 6^), GO:0007229 (“integrin-mediated signalling pathway”, p_adj_ = 1.6e^− 5^), and GO:0006955 (“immune response”, significant: 8; expected: 1.54; p_adj_ = 1.5e^− 4^). This dataset contains two genes encoding for an H-2 class II histocompatibility antigen chain: ENSONIG00000019943 and ENSONIG00000003904. Accession GO:0048854 (“brain morphogenesis”, significant: 2; expected: 0.15; p_adj_ = 0.001) was also significant enriched, however it is represented by only 2 genes: *atp1a1* (ENSONIG00000012456), encoding for ATPase Na+/K+ transporting subunit alpha 1a, and *shank3* (SH3 and multiple ankyrin repeat domains 3). Similar to inversions, we looked at GO enrichment across the phylogenetic tree (Additioal file 4: Supplementary File 3). While only one gene (*lyz*) was found in conserved duplications (after filtering for evidence of tandem repeats and presence in the Ensembl annotation), we had 41 genes for the *M. zebra*-*P. nyererei* subtree and 58 for the lineage including *H. burtoni* as well (Supplementary Fig. 3)*.* However, in all of these cases the significantly enriched terms were represented by very low (< 4) numbers of genes.

Altogether, our analyses provide the first insights into the possible contribution of SVs to the evolution of adaptive traits in African cichlids, including circadian rhythm, developmental processes and immune response mechanisms.

### Validation of selected deletion and inversion events

**Fig. 7:**
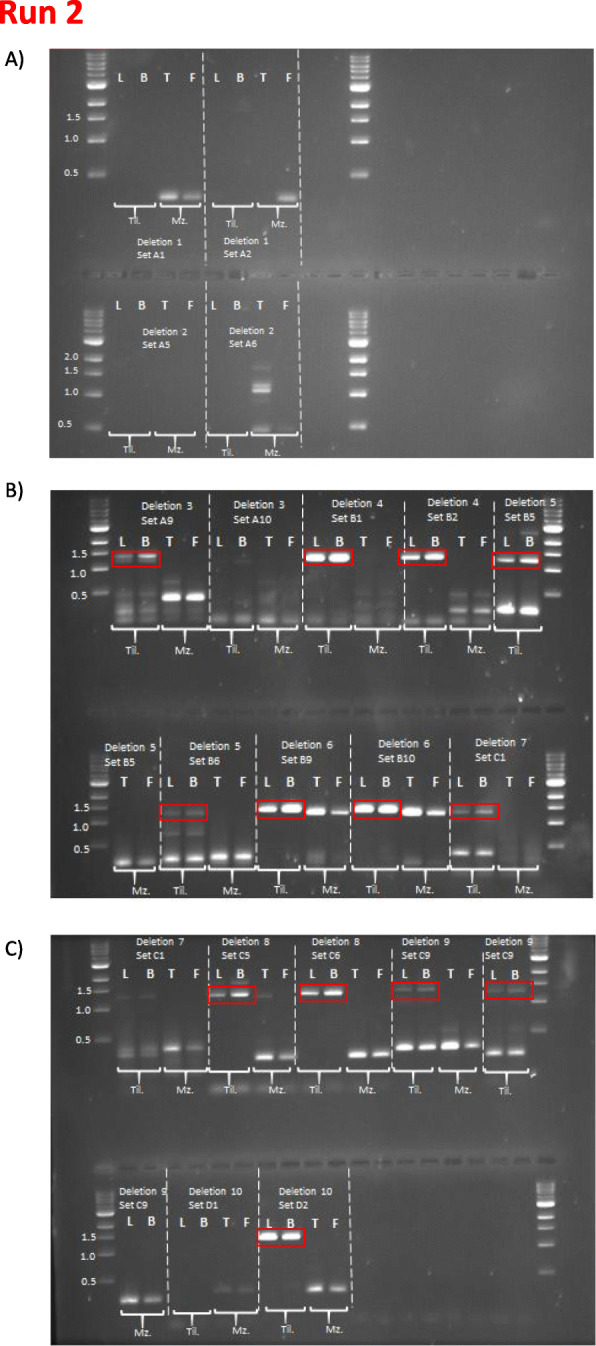
Gel images of PCR run 2 (validation of 10 deletion events in *M.zebra*). Red boxes indicate the expected product in the absence of the deletion (*O. niloticus* samples). **a** gel images for deletions 1 and 2. **b** results for deletions 3 to 7. C) images for deletions 7 to 10. No support was found for deletions 1,2 and 6. Key: L = liver, B = brain, T = Testis, F = fin, On = *O.niloticus*, Mz = *M. zebra*

**Fig. 8:**
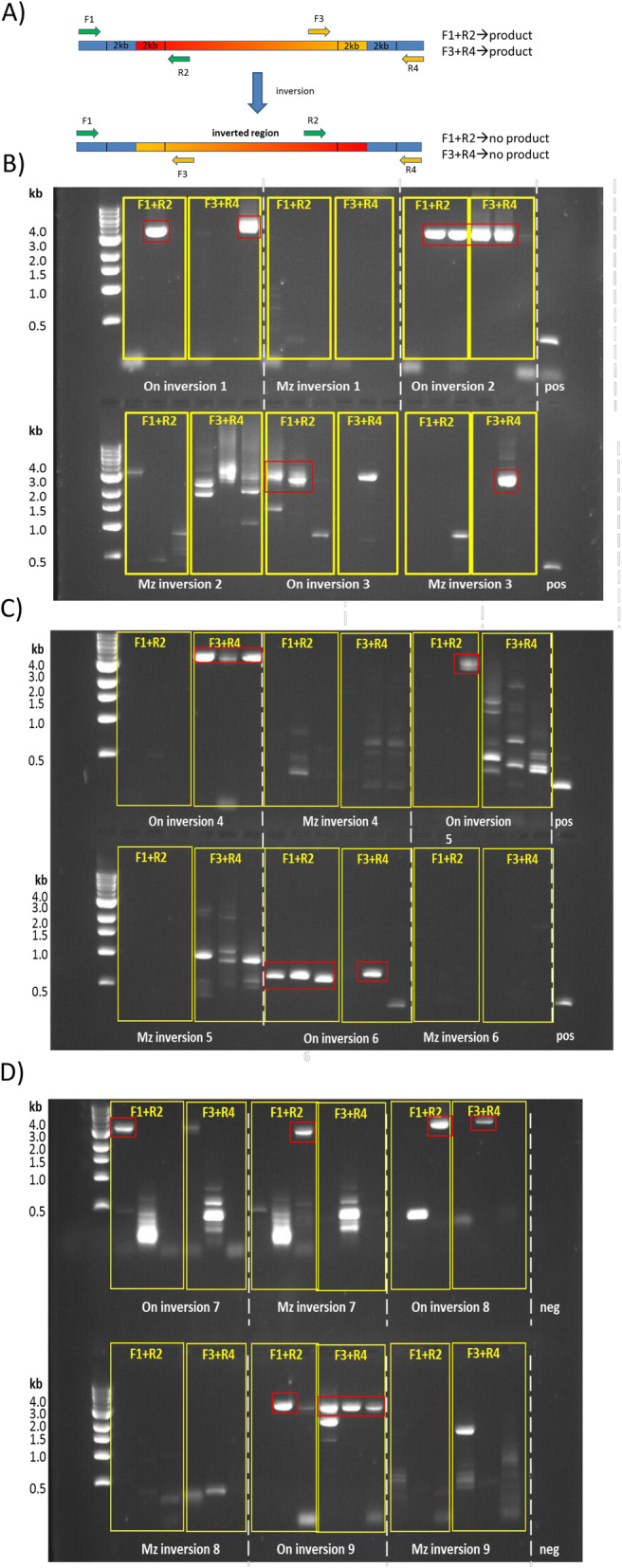
Gel images of PCR run 3 (validation of 9 inversion events in *M.zebra*). Red boxes indicate the expected product in the absence of the inversion (*O. niloticus* samples). **a** Schematic of the primer design (Primer set 1: F1 + R2; primer set 2: F3 + R4). The inverted region is labelled with a red to yellow gradient. **b** gel images for inversions 1 to 3. **c** images for inversions 4 to 6. **d** images for inversions 7 to 9. Limited or no support was observed for inversions 4, 5 and 7. Key: On = *O.niloticus*, Mz = *M. zebra*

In order to better understand the reliability of our computational analyses, we decided to validate selected deletions and inversions by PCR amplification of the rearranged genomic region (Figs. 7 and 8, Supplementary Fig. 4, Methods). We first focused on 10, medium sized (1-5 kb) deletion events annotated in *M. zebra* (Table 2). For the validation, we compared experimental results obtained using tissue samples for *M. zebra* (liver and brain) and *O. niloticus* (testis and fin). In this comparison, *O. niloticus* represents the SV-free reference sequence, while *M. zebra* is predicted to carry the deletion event (and hence show a smaller amplification product). Figure 7 provides an overview of the results of the second PCR run. We could confidently confirm the deletion event in 7 out of 10 cases. For deletions 1 and 2, we were not able to detect the expected products. As for deletion 6, we had discordant results between run 1 (Supplementary Fig. 4) supporting the SV, and run 2 showing the expected product in both *M. zebra* and *O. niloticus*. Even excluding deletion 6, we obtained a 70% concordance between our computational predictions and the PCR validation, providing evidence for the reliability of our bioinformatics pipeline.

**Table 2:** Genomic coordinates and expected product size of the 10 tested deletions. DEL_1, DEL_2 and DEL_6 could not be confirmed by PCR

ID	genomic coordinates	Expected product size (kb): primer set 1 & 2
DEL_1	contig401:86756–90,467	**Set 1**: 3.3, **Set 2**: 3.3
DEL_2	contig429:64638–70,314	**Set 1**: 5.2, **Set 2**: 5.2
DEL_3	lg1:9504350–9,506,050	**Set 1:** 1.3, **Set 2:** 1.2
DEL_4	lg5:529562–531,357	**Set 1:** 1.3 **Set 2:** 1.4
DEL_5	lg11:29218518–29,220,314	**Set 1:** 1.3 **Set 2:** 1.3
DEL_6	lg12:27848949–27,850,738	**Set 1:** 1.4 **Set 2**: 1.4
DEL_7	lg16:40913013–40,914,775	**Set 1:** 1.3 **Set 2:** 1.3
DEL_8	lg17:27332603–27,334,390	**Set 1:** 1.3 **Set 2:** 1.3
DEL_9	lg18:23620664–23,622,449	**Set 1:** 1.3 **Set 2:** 1.3
DEL_10	lg20:8700540–8,702,339	**Set 1:** 1.3 **Set 2:** 1.3

Next, we adapted the primer design strategy for the validation of 9 selected inversions (Table 3), ranging from 1 kb to 10 Mb in size. We chose 7 events containing genes involved in either retina development (GO:0060041) or innate immune response (GO:0045087), plus 2 additional, smaller (< 4 kb) inversions. Different primer sets were designed to match sequences flanking either of the two breakpoints (Table 3, Methods). PCR results (Table 3, Fig. 8) confirmed the majority of these inversions, providing strong support for 6 events, partial support for 1 event (inv_4) and poor support for the remaining 2 (inv_5 and inv_7). Among the genes inside validated inversions (Additional file 5: Supplementary File 4), we find many genes involved in retina development: *sf1* (splicing factor 1) as part of inv_2; *vax2* (Ventral Anterior Homeobox 2) as part of inv_3; genes id2 (Inhibitor of Differentiation 2), *exoc5* (Exocyst Complex Component 5) and ppm1a (Protein Phosphatase 1A) located inside inv_8. No gene annotated to GO:0045087 (“innate immune response”), however, was found inside these validated inversions. Altogether, these results demonstrate the reliability of our bioinformatics analyses, and provide additional, experimental support to our inferences.

**Table 3:** Genomic coordinates and expected product size of the 10 tested deletions. INV_4, INV_5 and INV_7 could not be confirmed by PCR

ID	genomic coordinates	Expected product size (kb): primer set 1	Expected product size (kb): primer set 2
INV_1	lg1:23244839–23,247,964	3.6, 3.7, 3.9	3.4, 3.7, 3.8
INV_2	lg3a:742711–10,825,884	3.6, 3.6, 3.6	3.5, 3.7, 3.7
INV_3	lg3b:6690892–10,458,580	3.4, 3.8, 3.7	3.7, 4.1, 3.7
INV_4	lg9:24428050–24,471,026	3.7, 3.8, 3.3	3.8, 3.6, 3.9
INV _5	lg13:30450556–31,749,349	3.7, 3.5, 3.7	3.7, 3.6, 3.7
INV _6	lg15_10916855–10,917,869	0.7,0.7,0.6	0.7,0.7,0.3
INV _7	lg17:24916–1,818,230	3.6, 3.9, 3.4	3.8, 3.5, 3.9
INV _8	lg19:15124477–19,797,483	3.5, 3.6, 3.9	3.6, 3.6, 3.5
INV _9	lg20:7601421–8,565,000	3.3, 3.8, 3.6	3.6, 3.6, 3.6

## Discussion

Our work uncovers a new, important aspect of the adaptive radiation of East African cichlids. We demonstrate the presence of extensive structural rearrangements across representative species of the three Great Lakes, and strikingly, we show that these large-scale variants are likely implicated in the evolution of important adaptive traits.

We inferred the gain and loss patterns of all annotated SVs across the phylogenetic tree, thus identifying high proportions of lineage specific gains. While the size distributions are generally comparable across different conservation levels, we see a shift towards larger sizes in the case of deeply conserved inversions.

High proportions of lineage specific gains may provide novel opportunities for the adaptive evolution of these species. Therefore, we investigated the repertoires of genes affected by inversions and duplications, considering species specific and more conserved events separately. Among the most interesting biological processes associated with inversions, we find “behavior” (GO:007610), “retina development in camera-type eye” (GO:0060041), “pectoral fin development” (GO:0033339) and “embryonic skeletal system development” (GO:0048706). Moreover, we found enrichment for “neuron development” (GO:0048666) associated with events in the Haplochromine lineage (*M. zebra*, *P. nyererei*, *H. burtoni* is We can speculate that the enrichment for developmental processes reflects the implication of structural variation in shaping the great morphological and behavioural variation observed in East African cichlids [17–19, 52]. The enrichment for “retina development in camera-type eye” is however particularly striking, given that the adaptive radiation of East African cichlids is associated with the evolution of the visual system. This has been implicated in the adaptation to different water depths and turbidity conditions, as well as in female mating preferences [14, 16].

Accession GO:0033339 includes gene *Cyp26c1*, lying in a sex associated region identified in *H. burtoni* [53]. This result opens to speculations on the link between inversion events, suppressed recombination, and the divergence of sex-associated traits across lineages.

When we consider duplicated regions, we find an enrichment for “antigen processing and presentation” (GO:0019882) and additional immune related categories. This “theme” is common to both species-specific and multi-species events. However, when we consider different subtrees separately, the number of significant genes drops dramatically, making us less confident about the biological relevance of the enrichment results. Our set of genes inside duplicated regions include an “H-2 class II histocompatibility antigen” locus, as well as *ilf2* (interleukin enhancer binding factor). The observed association between immune genes and duplication events is not surprising, being in line with previous studies on the fast, adaptive evolution of the vertebrate immune system [54–56]. In cichlids, differences in parasite communities across foraging habitats can determine strong selective pressure, favoring adaptive phenotypes and ecological speciation. In particular, several studies have highlighted extensive variation in MHC pools which suggests immunogenetic adaptation [57–63]. Host-parasite coevolution in *Pseudotropheus fainzilberi* and *P. emmiltos* (a pair of sympatric Lake Malawi species) appear to have driven adaptive divergence in MHC alleles, affecting odor-mediated mate choice and leading to reproductive isolation [59]. A large scale analysis of MHC diversity across the major tribes of Lake Tanganyika cichlid fishes [9] showed how different cichlid tribes partially differ in both parasite communities and MHC diversity. The distinct MHC profile of the *Limnochromini*, for example, suggests that distinct immunogenetic properties are selected in deep water.

In the threespine stickleback (*Gasterosteus aculeatus*), it has been shown that Major Histocompitability Complex (MHC) genes are linked with female mating preference, suggesting that divergent selection acting on MHC genes might influence speciation [64, 65].

While our results strongly suggest that structural variation has been implicated in the adaptive evolution of African cichlids (especially for retina development and immune response), their interpretation in the light of morphological and ecological variation remains both challenging and speculative. Moreover, investigating the gene enrichment results in the light of ecological or morphological variation does not seem to highlight any clear pattern. Additional analyses and experiments would clearly be required in order to draw a link between gene enrichment analyses and the evolution of specific adaptive traits.

In order to gain more confidence on the results of our SV detection pipeline, we decided to validate selected events by PCR. These experiments provided strong support for 6 out of 9 inversions. The genomic regions of these 6 validated events include *sf1* (splicing factor 1), *vax2* (Ventral Anterior Homeobox 2) and other genes which are involved in retina development. Thus, we were able to provide additional, experimental support for selected SV events, potentially involved in the adaptive evolution of the visual system.

Additional PCR experiments also confirmed 7 deletion events out of 10 tested. This suggests similar levels of accuracy (~ 70%) in the SV identification method for deletions and inversions.

It is important to mention that we did not sequence the PCR products to confirm the amplification of the target regions. We are aware that this represents a limitation of our study. However, we would like to stress some important points: 1) our primer design was based on the high quality PACBIO reference of *O. niloticus*; 2) all validated SVs show a clear and strong correlation between the strongest PCR band and the expected product size; 3) in all validated SVs, the pattern is perfectly and uniquely consistent with the hypothesis of a deletion (or inversion) occurring in *M. zebra* as compared to the *O. niloticus* reference; 4) in the case of deletions, our validation is supported by technical replicates that lead to the same result.

We also investigated the possibility of differential evolutionary patterns between inverted and non-inverted regions by comparing their repetitive element landscapes in *M. zebra*. Despite the observation of a significant enrichment in both DNA transposons and LTR elements, we observed little difference in repeat content. This holds true when comparing duplicated regions to the rest of the genome.

## Conclusions

In this study, we provide a comprehensive overview of rearrangement evolution in East African cichlids, and highlight their likely contribution to the evolution of adaptive traits.

The results presented here are likely to inspire further studies, focusing on several aspects of rearrangement evolution. These might include: the evolution of genome size in East African cichlids; the contribution of inversions to speciation events, as highlighted by previous studies [36–39]; the role of SVs in shaping the expression landscape by altering gene sequences, gene copy number, or regulatory elements [66–69]; further studies on SV identification, evolution and biological role, considering a different (and possibly greater) set of species. The inclusion of data from additional species, and the resolution of intra and inter specific variability would result in a much greater power in reconstructing the evolutionary dynamics of each SV event [33, 70]. Moreover, it would facilitate the identification of any association between SVs and traits under selection.

The evolution of cichlids in African lakes represents an impressive example of how a relatively low degree of genetic variation can provide the substrate for an explosive and rapid species radiation, allowing for the adaption to many different ecological niches. Single nucleotide variants, large scale rearrangements, transposable elements and several regulatory mechanisms can all contribute to the evolution of diverse genetic traits with high adaptive potential. We are only starting to understand the evolutionary dynamics and molecular mechanisms underlying this impressive radiation, and much work is still needed to shed light on all the different aspects and key players involved.

## Methods

### SV calling

Paired-end libraries available for *Neolamprologus brichardi*, *Metriaclima zebra, Pundamilia nyererei* and *Haplochromis burtoni* [22] were downloaded using fastq-dump from the sra-toolkit (https://www.ncbi.nlm.nih.gov/sra/docs/toolkitsoft/). Due to lower base quality issues, the last 30 nt at the 3′ end of the longest, 100 nt reads were trimmed. All libraries were mapped against the *O. niloticus* genome assembly (Supplementary Fig. 1) using gmap [71]. The resulting bam files were sorted and indexed using samtools [72], then used as input for 3 algorithms: Breakdancer [42], Delly [43] and Pindel [44].

SV predictions were first filtered by: a minimum of 2 libraries and 5 discordantly mapping read pairs supporting the call (Breakdancer and Pindel); a Breakdancer score of 99; both a PASSED and PRECISE flag provided by Delly’s output files. For each tool and rearrangement class separately (with the exception of translocations), we merged predictions with a reciprocal coordinate intersection of at least 90% into a single SV call.

The sets of merged filtered calls of each algorithm were then compared in a pairwise manner. Specifically, we used Bedtools intersect (Quinlan 2014) to identify SVs independently called by two different algorithms, with a reciprocal intersection at least 90% of the SV region. This gave us three sets of SVs supported by at least 2 algorithms: Breakdancer+Delly, Breakdancer+Pindel and Delly+Pindel (Supplementary Fig. 5). The annotations of each SV class across all species was then combined into a single BED file. For each combined set, we then carried out a conditional merging of the SV genomic coordinates. For events up to 0.5 kb in size, we required a minimum of 50% reciprocal intersection for multiple calls to be merged together. For size ranges of 0.5-1 kb, 1–10 kb and all events greater than 10 kb, we used a threshold 80, 90 and 95%, respectively (in each range, we included the bottom value while excluding the top one).

### Overlap analyses and GO:term enrichment

Analyses of overlap between SVs and genome annotation were performed using GAT [73]. The *O_niloticus* UMD1 gene annotation was downloaded from the NCBI database and converted into BED format. Genes inside SV regions were identified by comparing the gene annotation with the genomic coordinates of our SV dataset, using Bedtools intersect (Quinlan 2014). We selected genes fully contained inside an SV event by using the options “-a genes.bed -b SV.bed -f 1”.

We used a combination of Biomart (www.ensembl.org/biomart/martview/) and DAVID (https://david.ncifcrf.gov/) to map all NCBI gene ids to the corresponding Ensembl gene ids.

GO:term enrichment was performed on the set of genes mapping to an Ensembl gene ids. We used the elim algorithm from the R package TopGO [74]. The gene background was defined as the set of all genes in the NCBI annotation mapping to an Ensembl gene id.

### Whole genome alignments and repeat content analyses

In order to generate whole genome alignments between the latest *M. zebra* [75] and *O. niloticus* [41] assemblies, we ran Satsuma2 (https://github.com/bioinfologics/satsuma2) using the following parameters: *-slaves 10 -threads 16 -km_mem 120 -sl_mem 120 -prob_table true -min_prob 0.99999 -min_seed_length 20 -max_seed_kmer_freq 1 -min_matches 10 -dump_cycle_matches 1.*

In order to compare Satsuma2 results with our dataset of *M. zebra* deletions (*O. niloticus* genomic coordinates), we converted the satsuma_summary.chained.out output file into a 6 columns BED file. We then used the command “bedtools intersect” from Bedtools [76] to identify alignments of sequences overlapping a deletion event. A deletion was considered to be discordant with Satsuma2 if at least one alignment spanning 50% or more of the predicted deleted region could be identified.

For the analysis of the repeat content inside and outside SV regions, the software RepeatMasker was run on the *O.niloticus* reference to identify repetitive elements genome-wide. The .out result file of RepeatMasker was reformatted to generate a 6 column BED file. For each SV separately, we then used Bedtools intersect [76] to identify repeat elements fully contained inside the SV regions. The analyses were restricted to the SVs annotated in *M. zebra.* Overlapping repeat elements were separated based on the percentage of divergence from the consensus sequence, provided in the .out result file. An equivalent approach was used to identify repeat elements outside SV events. The repeat content was then calculated as the proportion of repeat nucleotide positions over the total length of the genomic space considered (total size of SV space or the genomic space outside SVs).

### Experimental animals

The *M. zebra* individuals were maintained in the cichlid fish facility at University of Hull managed by Alan M. Smith and Domino Joyce. In order to maintain a healthy colony and stimulate breeding and good quality egg production throughout the year, *M. zebra* individuals were kept under optimal conditions. The *O. niloticus* individuals are lab-acclimated Egyptian strains (Lake Manzala stock originally maintained at Swansea and Stirling University) kept in the Tilapia fish facility at ARO and managed by Avner Cnaani. In order to maintain a healthy colony and stimulate breeding and good quality egg production throughout the year, *O. niloticus* individuals were kept under optimal conditions which was, in this case, a temperature of 25C, pH 7.9 and salinity of 0.02%.

*M. zebra* individuals were sacrificed according to Home Office license schedule 1, killing using overdose of MS-222 (tricaine) at the lab of Dr. Domino Joyce, The University of Hull, UK. *O. niloticus* individuals were sacrificed according to IACUC certification by the Israeli Ministry of Health’s Council for Experimentation on Animals, licensed schedule 1, killing using overdose of MS-222 (tricaine) at the lab of Dr. Avner Cnaani, Institute of Animal Science, Agricultural Research Organization (ARO), Bet Dagan, Israel. Upon sacrifice, relevant tissues were dissected and preserved in either RNAlater or laboratory grade absolute ethanol (EtOH). A completed ARRIVE guidelines checklist is included as Additional file 6: Supplementary File 5.

### PCR validation of structural variants

Oligonucleotide primers were designed against the latest *O. niloticus* assembly using Primer3 [77] and all primers were synthesised by Integrated DNA Technologies, Iowa.

DNA was extracted from samples of frozen tissue or tissues preserved in ethanol (25 mg) from 1 *M. zebra* individual (fin, testis) and 1 *O. niloticus* individual (brain, liver), using the MagAttract HMW DNA Kit (Qiagen, CA) according to the manufacturer’s protocol for “Manual Purification of High Molecular Weight Genomic DNA from Fresh or Frozen Tissue”. Final DNA concentrations were determined using Qubit fluorometer™ 2.0 (Invitrogen, Life Technologies) and purity was assessed using A260:280 ratio (≥1.8) by measurement on a Nanodrop™ spectrophotometer (Thermofisher Scientific). PCR products were amplified according to manufacturer’s protocol for NEBNext® High-Fidelity 2X PCR Master Mix in 25 μl reactions using 50 ng of DNA template. PCR cycle conditions were followed as stated in manufacturer’s protocol and extension times were adjusted according to length of expected product size. PCR products were visualised on 1.5% (w/v) agarose gels stained with SYBR™ Safe DNA Gel Stain and imaged using the Alliance 2.7 gel documentation system (UVITEC, Cambridge).

## Supplementary information

**Additional file 1: Supplementary Figure S1.** Schematic of the SV detection pipeline. **Supplementary Figure S2.** Association of different enriched GO terms across the phylogenetic tree, considering the genes found inside inverted regions (up to 5 Mb). For each node, selected GO terms are shown for the inversion events specific to (and conserved across) the *M.zebra + P.nyererei* lineage (top right), *M.zebra + P.nyererei + H.burtoni* lineage (top-centre) and conserved across all species (bottom left). Numbers on the top of each bar indicate the number of observed genes. **Supplementary Figure S3.** Association of different enriched GO terms across the phylogenetic tree, considering the genes found inside duplicated regions. For each node, selected GO terms are shown for the duplication events specific to (and conserved across) the *M.zebra + P.nyererei* lineage (top right), the *M.zebra + P.nyererei + H.burtoni* lineage (top-centre) and conserved across all four species (bottom left). Numbers on the top of each bar indicate the number of observed genes. **Supplementary Figure S4.** A) Experimental design for the PCR validation of deletion events. Arrows represent primer sequences mapped to the genomic sequence (in blue and red). Primer couple AF1 + AR1 is used to test for the presence or absence of the deletion event (expected to differ by about *N* bp in the amplification product). Primer couples BF1 + BR1 and CF1 + CR1 are used as a control (expected product:300-400 bp). B-E gel images of PCR run 1, used for the validation of 10 deletion events. See Fig. 7 for a detailed explanation of the figure labels. **Supplementary Figure S5.** Venn *diagram* depicting the intersection between filtered SV calls of all three tools (*Breakdancer*, *Delly* and *Pindel*). In the case of insertions, no intersection was found between *Breakdancer* and the other two tools.

**Additional file 2: Supplementary file 1.** Genomic coordinates and annotation across species for all SV classes.

**Additional file 3: Supplementary file 2.**Results of MW test to compare SV size distribution across different conservation categories. For each comparison, the *p*-value is indicated. In the case of a significant difference, the directionality of the change is indicated. For example, “1 < 2” indicates that the ranks of the 1-species dataset are significantly lower than those for the 2-species dataset.

**Additional file 4: Supplementary file 3.** Names and corresponding GO annotation for different subsets of genes inside duplicated and inverted regions

**Additional file 5: Supplementary file 4.** Genes found inside PCR validated inversions. Each row corresponds to one gene. Genomic coordinates of the associated inversion, and the number of species carrying the inversion are indicated, along with the gene name and ncbi id.

**Additional file 6: Supplementary file 5.** ARRIVE checklist.

## Data Availability

The data set supporting the results of this article is available in the Short Read Archive (SRA) repository (https://www.ncbi.nlm.nih.gov/sra) under the following accessions: PRJNA59571 (SRP004171) for *O. niloticus*; PRJNA60365 (SRP004799) for *N. brichardi*; PRJNA60367 (SRP004869) for *P. nyererei*; PRJNA60369 (SRP004788) for *M. zebra*; and PRJNA60363 (SRP004787) for *H. burtoni* [22].

## Abbreviations

SV: Structural Variant
LTR: Long Terminal Repeat
SINE: Short Interspersed Nuclear Element
PCR: Polymerase Chain Reaction
ARRIVE: Animal Research: Reporting of In Vivo Experiments
