# Supplementary material for: Analysis of structural variants in four African cichlids highlights an association with developmental and immune related genes
Source: BMC Evol Biol. 2020 Jun 22;20:69. doi: 10.1186/s12862-020-01629-0 (PMC7309985; doi:10.1186/s12862-020-01629-0)
Supplement: Supplementary file 1 — Additional file 1: Supplementary Figure S1. Schematic of the SV detection pipeline. Supplementary Figure S2. Association of different enriched GO terms across the phylogenetic tree, considering the genes found inside inverted regions (up to 5 Mb). For each node, selected GO terms are shown for the inversion events specific to (and conserved across) the M.zebra + P.nyererei lineage (top right), M.zebra + P.nyererei + H.burtoni lineage (top-centre) and conserved across all species (bottom left). Numbers on the top of each bar indicate the number of observed genes. Supplementary Figure S3. Association of different enriched GO terms across the phylogenetic tree, considering the genes found inside duplicated regions. For each node, selected GO terms are shown for the duplication events specific to (and conserved across) the M.zebra + P.nyererei lineage (top right), the M.zebra + P.nyererei + H.burtoni lineage (top-centre) and conserved across all four species (bottom left). Numbers on the top of each bar indicate the number of observed genes. Supplementary Figure S4. A) Experimental design for the PCR validation of deletion events. Arrows represent primer sequences mapped to the genomic sequence (in blue and red). Primer couple AF1 + AR1 is used to test for the presence or absence of the deletion event (expected to differ by about N bp in the amplification product). Primer couples BF1 + BR1 and CF1 + CR1 are used as a control (expected product:300-400 bp). B-E gel images of PCR run 1, used for the validation of 10 deletion events. See Fig. 7 for a detailed explanation of the figure labels. Supplementary Figure S5. Venn diagram depicting the intersection between filtered SV calls of all three tools (Breakdancer, Delly and Pindel). In the case of insertions, no intersection was found between Breakdancer and the other two tools. [file 12862_2020_1629_MOESM1_ESM.pdf]

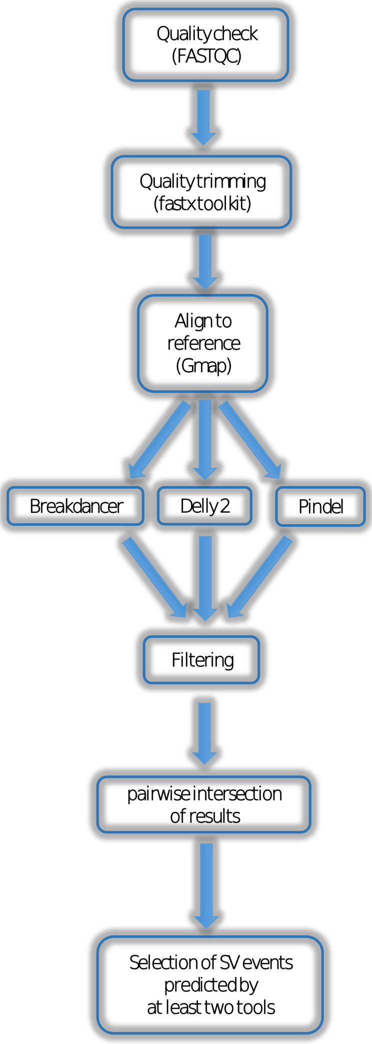

Supplementary Figure 1. Schematic of the SV detection pipeline

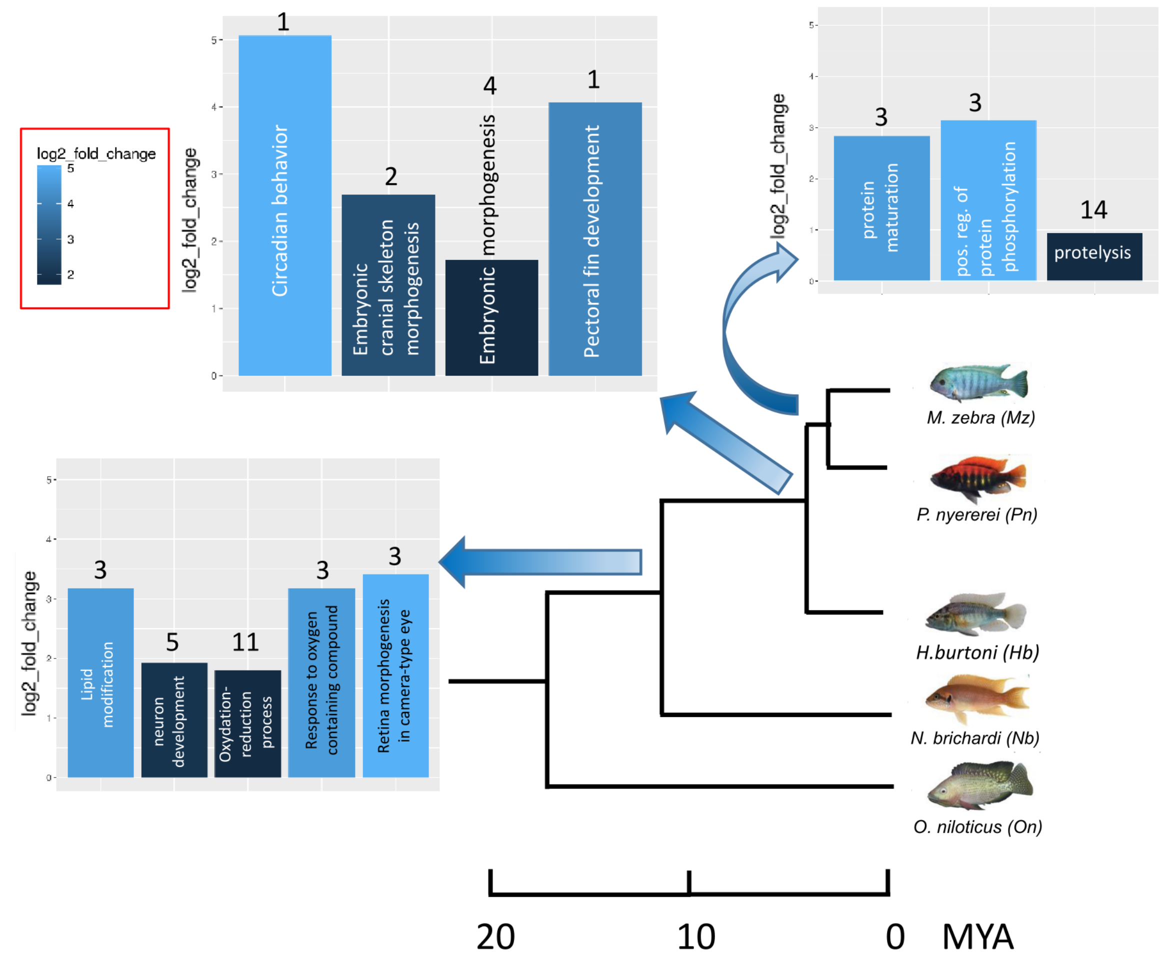

Supplemetary Figure 2. Association of different enriched GO terms across the phylogenetic tree, considering the genes found inside inverted regions (up to 5Mb). For each node, selected GO terms are shown for the inversion events specific to (and conserved across) the *M.zebra+P.nyererei* lineage (top right), *M.zebra+P.nyererei+ H.burtoni* lineage (top-centre) and conserved across all species (bottom left). Numbers on the top of each bar indicate the number of observed genes.

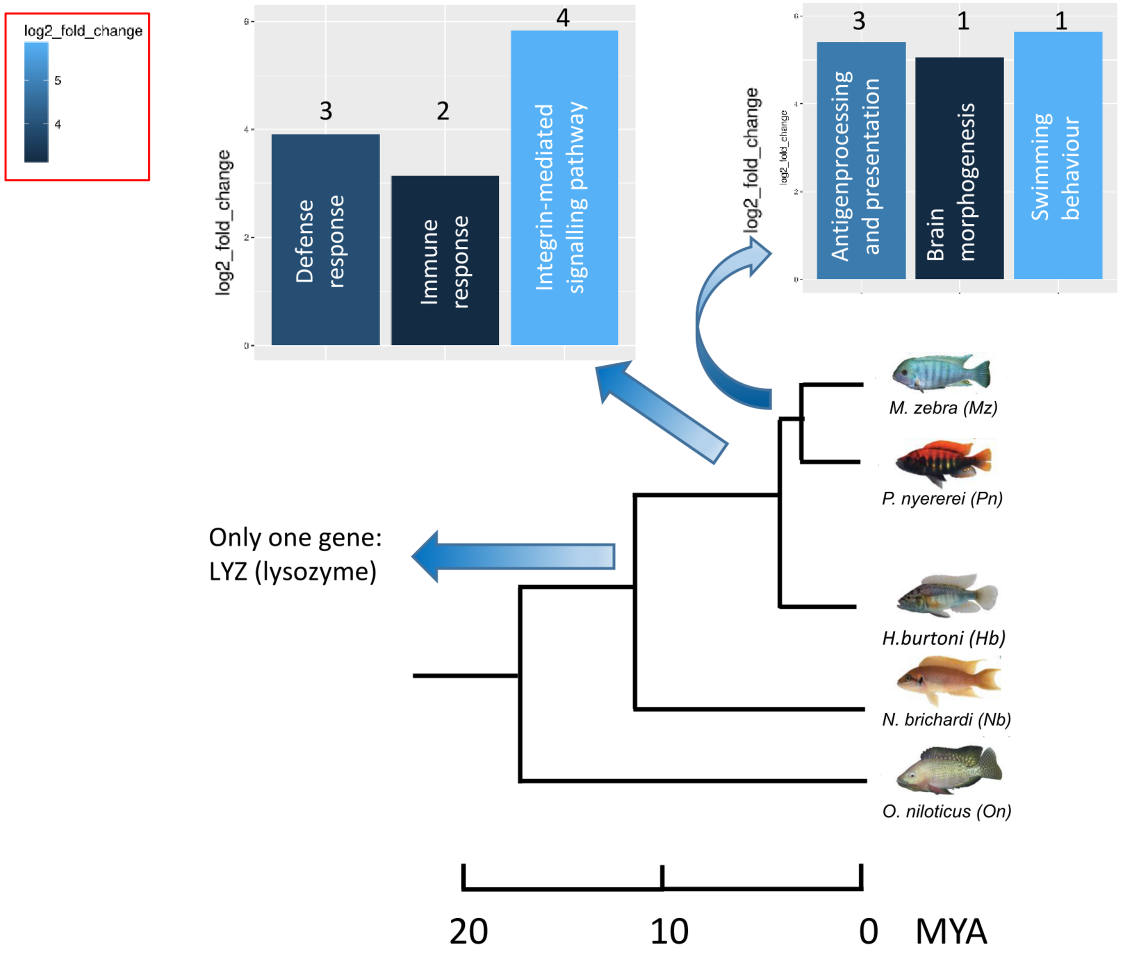

Supplementary Figure 3. Association of different enriched GO terms across the phylogenetic tree, considering the genes found inside duplicated regions. For each node, selected GO terms are shown for the inversion events specific to (and conserved across) the *M.zebra+P.nyererei* lineage (top right), *M.zebra+P.nyererei+ H.burtoni* lineage (top-centre) and conserved across all species (bottom left). Numbers on the top of each bar indicate the number of observed genes.

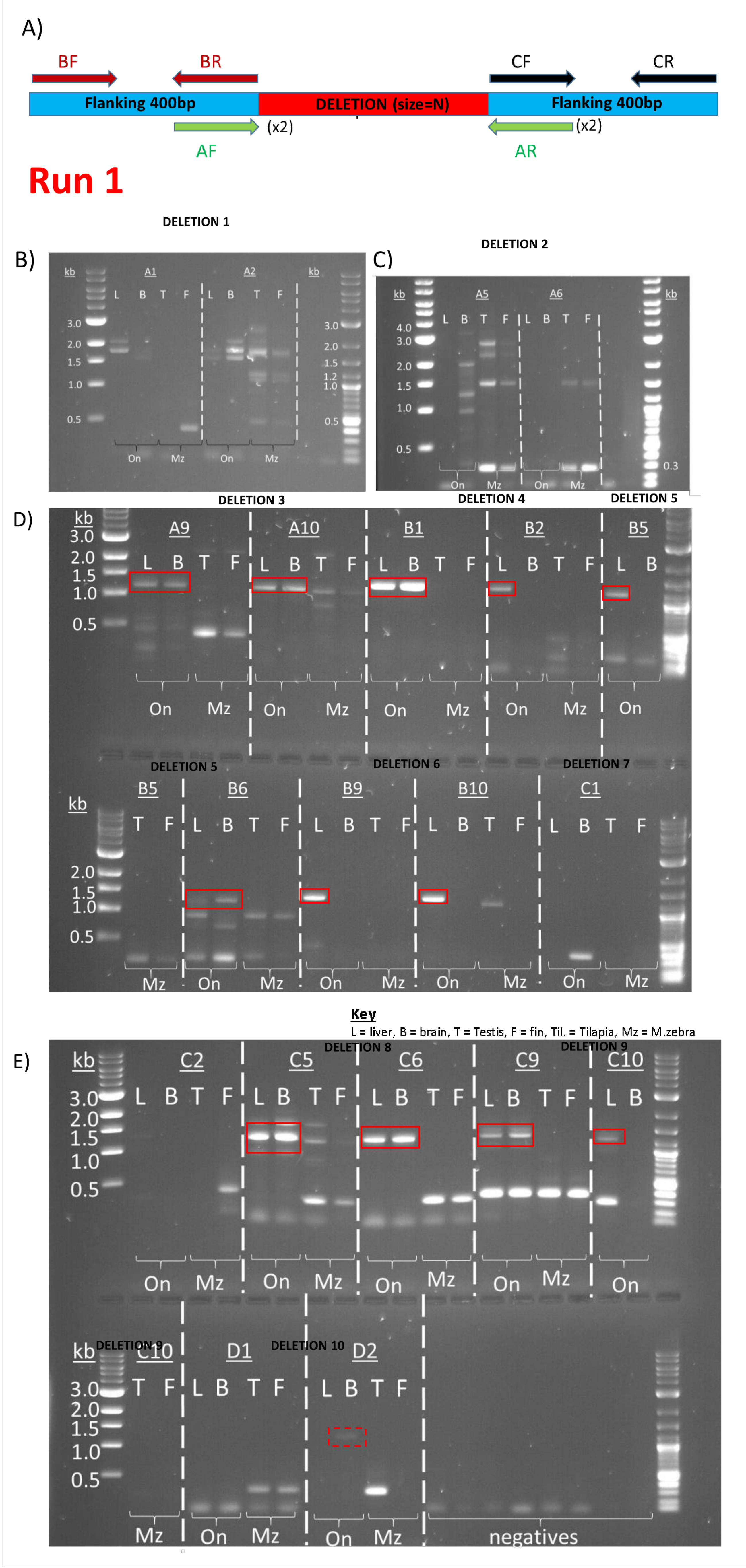

Supplementary Figure 4.A) Experimental design for the PCR validation of deletion events. Arrows represent primer sequences mapped to the genomic sequence (in blue and red). Primer couple AF1 +AR1 is used to test for the presence or absence of the deletion event (expected to differ by about N bp in the amplification product). Primer couples BF1 + BR1 and CF1 + CR1 are used as a control (expected product:300-400bp). B-E gel images of PCR run 1, used for the validation of 10 deletion events. See Fig. 7 for a detailed explanation of the figure labels

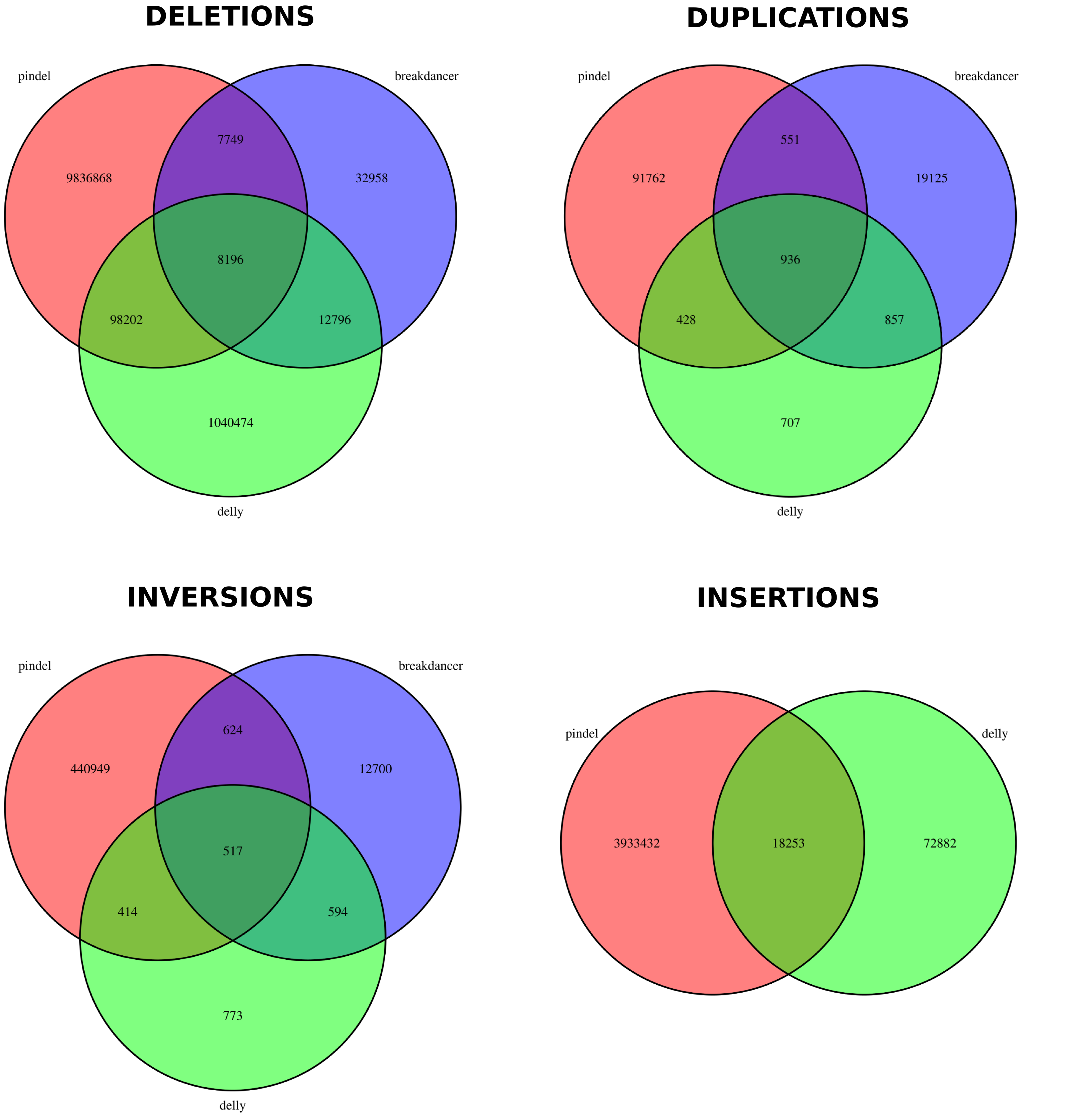

Supplementary Figure 5. Venn diagram depicting the intersection between filtered SV calls of all three tools (*Breakdancer*, *Delly* and *Pindel*). In the case of insertions, no intersection was found between *Breakdancer* and the other two tools.
