# Supplementary material for: Analysis of structural variants in four African cichlids highlights an association with developmental and immune related genes
Source: BMC Evol Biol. 2020 Jun 22;20:69. doi: 10.1186/s12862-020-01629-0 (PMC7309985; doi:10.1186/s12862-020-01629-0)

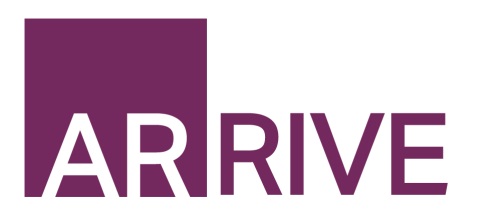


The ARRIVE Guidelines Checklist

Animal Research: Reporting In Vivo Experiments

Carol Kilkenny^1^, William J Browne^2^, Innes C Cuthill^3^, Michael Emerson^4^ and Douglas G Altman^5^

*^1^The National Centre for the Replacement, Refinement and Reduction of Animals in Research, London, UK, ^2^School of Veterinary Science, University of Bristol, Bristol, UK, ^3^School of Biological Sciences, University of Bristol, Bristol, UK, ^4^National Heart and Lung Institute, Imperial College London, UK, ^5^Centre for Statistics in Medicine, University of Oxford, Oxford, UK.*

|  | | ITEM | RECOMMENDATION | Section/ Paragraph |
| --- | --- | --- | --- | --- |
| 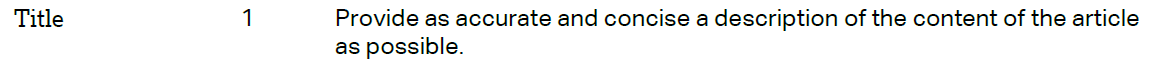 | | | Title |  |
| 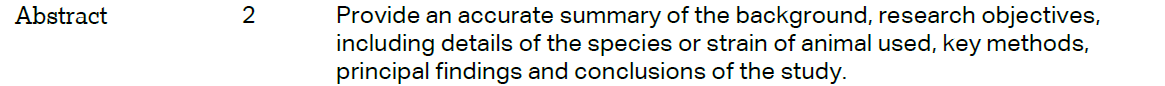 | | | Abstract |  |
| INTRODUCTION | | |  |  |
| 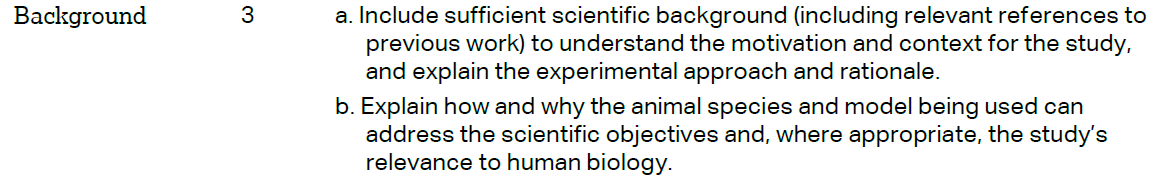 | | | Paragraph 1 |  |
| 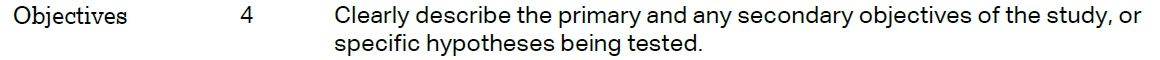 | | | Paragraph 5 |  |
| METHODS | | |  |  |
| 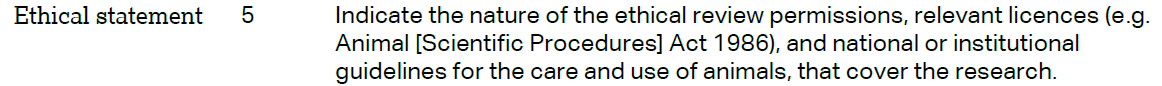 | | | Section “PCR validation of structural variants” |  |
| 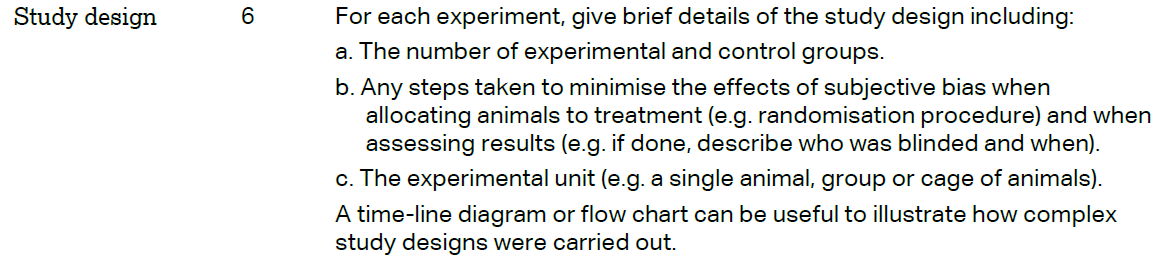 | | | Section “PCR validation of structural variants” |  |
| 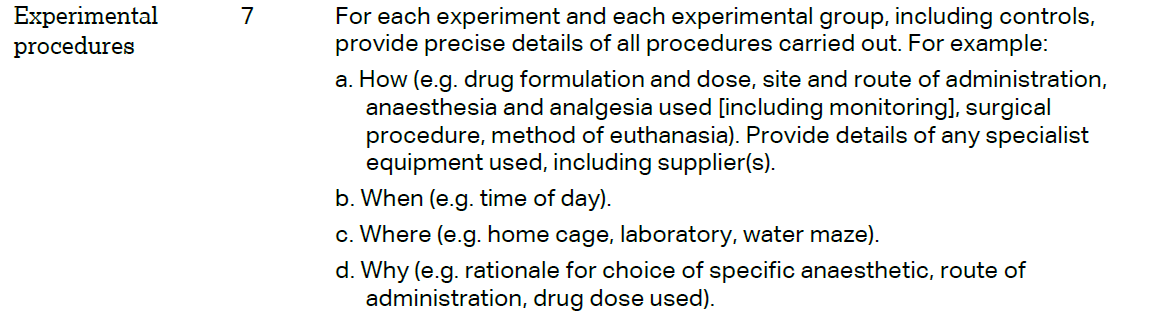 | | | Sections “PCR validation of structural variants”,  “Experimental animals” |  |
| 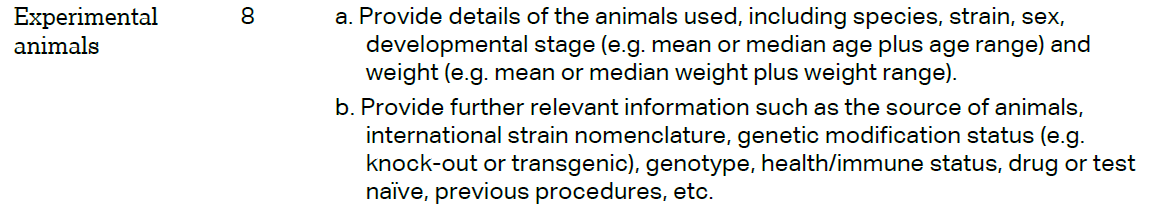 | | | Section ” Experimental animals” |  |

The ARRIVE guidelines. Originally published in *PLoS Biology*, June 2010^1^

| 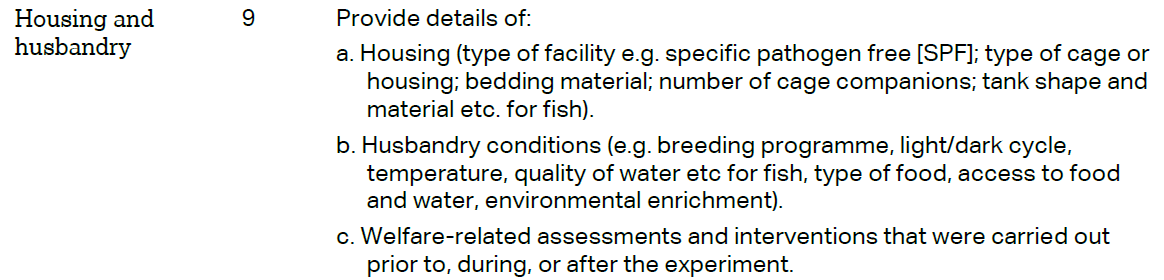 | Section ” Experimental animals” | |
| --- | --- | --- |
| 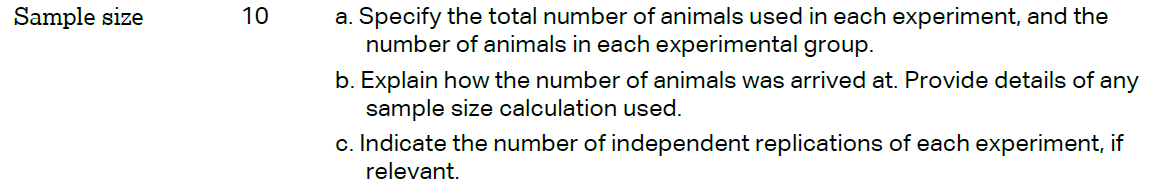 | Section “PCR validation of structural variants” | |
| 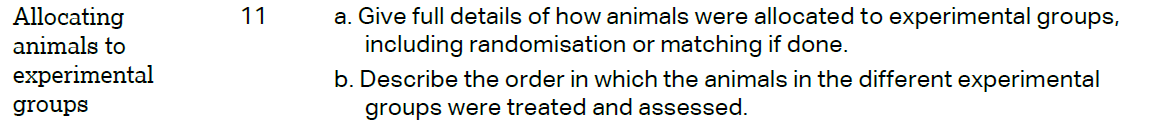 | Section “PCR validation of structural variants” | |
| 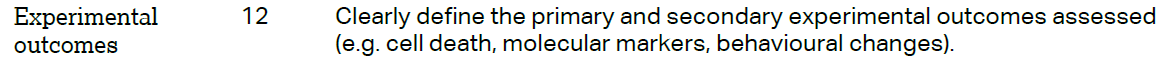 | Results, Discussion | |
| 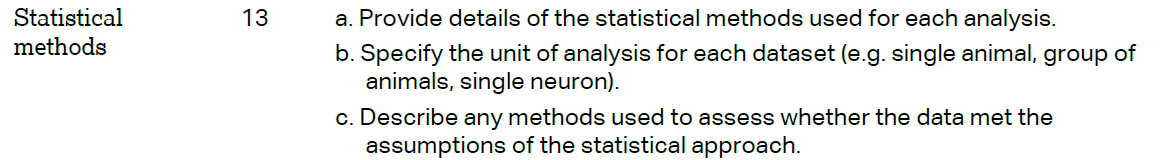 | Throughout the Methods | |
| RESULTS |  | |
| 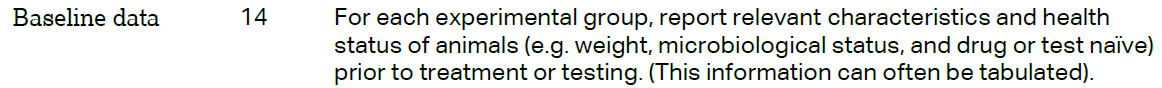 | Section “PCR validation of structural variants” | |
| 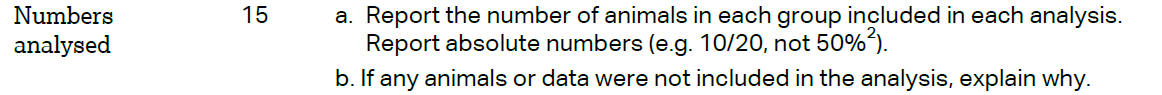 | Section “PCR validation of structural variants” | |
| 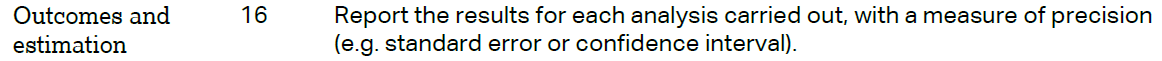 | Results | |
| 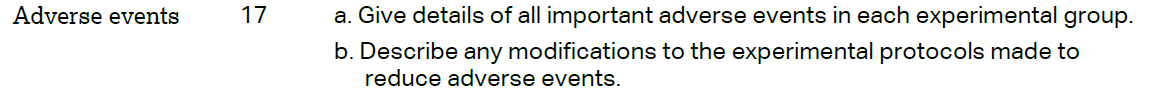 | Section “PCR validation of structural variants” | |
| DISCUSSION |  | |
| 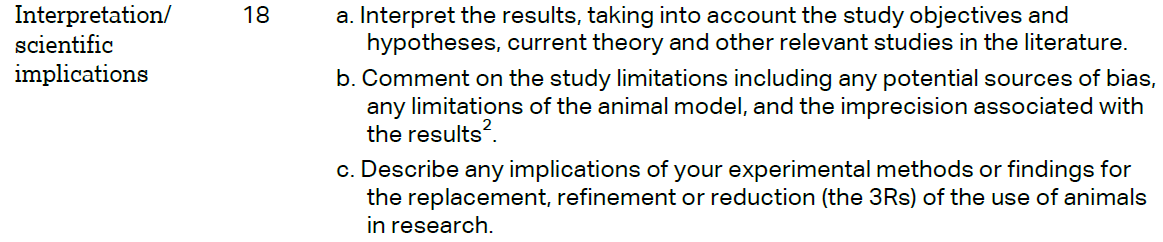 | Paragraphs 1-9 | |
| 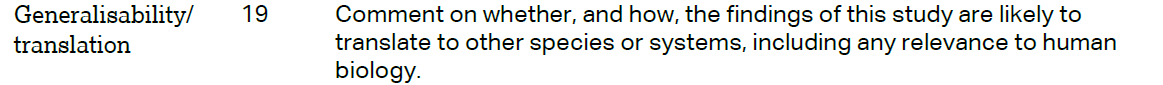 | Paragraphs 8-9 | |
| 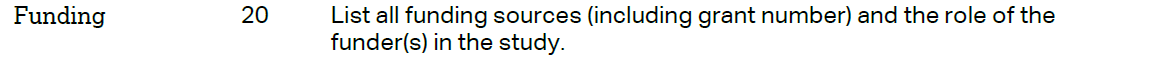 | | Section “ Declarations” |


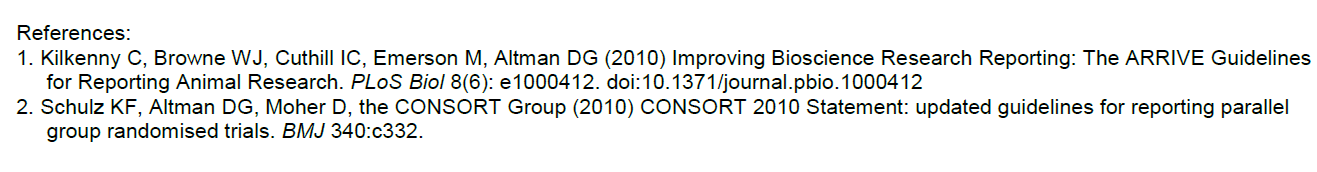

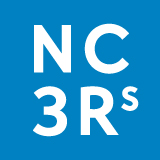

Supplement: Supplementary file 6 — Additional file 6: Supplementary file 5. ARRIVE checklist. [file 12862_2020_1629_MOESM6_ESM.docx]
